# Frequency of Autoantibodies and Connective Tissue Diseases in Chinese Patients with Optic Neuritis

**DOI:** 10.1371/journal.pone.0099323

**Published:** 2014-06-20

**Authors:** Hongyang Li, Yan Zhang, Zuohuizi Yi, Dehui Huang, Shihui Wei

**Affiliations:** 1 Department of Ophthalmology, The Chinese People’s Liberation Army General Hospital, Beijing, China; 2 Department of Ophthalmology, The General Hospital of Beijing Military Region, Beijing, China; 3 Department of Ophthalmology, The People’s Hospital Affiliated Wuhan University, Wuhan, China; 4 Department of Neurology, The Chinese People’s Liberation Army General Hospital, Beijing, China; Innsbruck Medical University, Austria

## Abstract

**Background:**

Optic neuritis (ON) is often associated with other clinical or serological markers of connective tissue diseases (CTDs). To date, the effects of autoantibodies on ON are not clear.

**Purpose:**

To assess the prevalence, clinical patterns, and short outcomes of autoantibodies and Sjögren’s syndrome (SS) involvement in Chinese ON patients and evaluate the relationship between ON, including their subtypes, and autoantibodies.

**Methods:**

A total of 190 ON patients were divided into recurrent ON (RON), bilateral ON (BON), and isolated monocular ON (ION). Demographic, clinical, and serum autoantibodies data were compared between them with and without SS involvement. Serum was drawn for antinuclear antibody (ANA), extractable nuclear antigen antibodies (SSA/SSB), rheumatoid factor (RF), anticardiolipin antibodies (ACA), and anti-double-stranded DNA antibody (A-ds DNA), anticardiolipin antibody (ACLs), anti-β2-glycoprotein I (β2-GPI) and Aquaporin-4 antibodies (AQP4-Ab). Spectral-domain optical coherence tomography (SD-OCT) was used to evaluate the atrophy of the optic nerve.

**Results:**

68 patients (35.79%) had abnormal autoantibodies, 26(13.68%) patients met diagnostic criteria for CTDs, including 15(7.89%) patients meeting the criteria for SS. Antibodies including SSA/SSB 23 (30.26%) (p1 and p 2<0.001) and AQP4–Ab10 (13.16%) (p1 = 0.044, p2 = 0.01) were significantly different in patients in the RON group when compared with those in the BON (P1 = RON VS ION) and ION (p2 = RON VS ION) groups. SS was more common in RON patients (p1 = 0.04, p2 = 0.028). There was no significant difference between SSA/SSB positive and negative patients in disease characteristics or severity. Similar results were obtained when SS was diagnosed in SSA/SSB positive patients.

**Conclusion:**

RON and BON were more likely associated with abnormal autoantibodies; furthermore, AQP4 antibody, SSA/SSB and SS were more common in the RON patients. AQP4 antibodydetermination is crucial in RON patients who will develop NMO. However, when compared with other autoantibodies, SSA/SSB detected in patients was not significantly associated with disease characteristics or severity.

## Introduction

Optic neuritis (ON) is an inflammatory optic nerve injury, which causes acute or subacute onset of vision loss in children and young adults [1]. Some patients experience recurrent episodes or bilateral ON occurring at the same time [2]. ON may be the first symptom of a central nervous system demyelinating and systemic disease, such as multiple sclerosis (MS) and neuromyelitis optica (NMO). Patients with NMO or MS often have accompanying autoantibodies and autoimmune diseases [3], [4], most commonly, but not limited to, Sjögren syndrome (SS) or a related profile of autoantibodies including antinuclear antibody (ANA), extractable nuclear antigen antibodies (SSA/SSB), rheumatoid factor (RF), anticardiolipin antibodies (ACA), and anti-double-stranded DNA antibody (A-ds DNA) [5] and AQP4 antibody. For these patients, a glucocorticoid treatment would not be the best therapeutic strategy. A treatment for autoimmune disease would be more important.

ON is an inflammatory demyelinating disease. Furthermore, in recent studies bilateral ON combined with SLE/SS cases has been reported, and this tape of ON has been considered more likely combined with AQP-4 antibody or relapse to NMO. [6], [7]. ON with autoimmune diseases present a relapsing remitting clinical profile, or lack of response to the regular glucocorticoid treatment [8]. The long-term visual prognosis is more severe in chronic relapsing inflammatory optic neuritis (CRION) patients and neuromyelitis optica-immunoglobulin G (NMO-IgG)-positive patients [9]. Thus, the understanding of frequencies and the various effects of autoantibodies or CTDs in ON patients is deemed crucial. Although some studies have reported the frequencies of ANA, SSA/SSB, RF, ACLs, and A-ds DNA in MS and NMO [10]–[12], with frequencies of SSA/SSB being higher than the others, data in ON are still missing. NMO patients require different treatment compared to patients with MS. Therefore early differentiation is very important [13]. AQP4 IgG antibodies are important in NMO as a high specificity in NMO [14]. AQP4 Ab was included in the revised diagnostic criteria for NMO, due to its very high specificity in NMO. AQP4 Ab is useful in predicting the severity of the disease course and probability of conversion to NMO at the first episode of isolated ON [14]. However, as AQP4-Ab were discovered only a few years ago, many previous studies were based on relatively small patient numbers [15]. There are few reports studying AQP4 antibody seropositivity in patients with clinically isolated syndrome (CIS) manifesting as different tape of ON [16], [17].

In this study, we evaluated the frequencies of autoantibodies in an ON population, including subtypes, to assess whether the presence of different autoantibodies had any clinical significance, and determine whether SSA/SSB and SS were more common in RON patients or not. Resolving this issue may play an important role in the development of diagnostic methods and therapeutic agents for improved treatment strategies for ON.

## Materials and Methods

Patients with ON were recruited from the ophthalmology department of The Chinese People’s Liberation Army General Hospital (PLAGH). Recruitment took place from November 2010 to April 2013, and patients meeting the inclusion criteria were offered participation in the study involving consultation and follow-up outpatient visits. The diagnosis of ON was confirmed using the optic neuritis treatment trial (ONTT) [18]. Patients who were included in our database fulfilled the following criteria:

- Acute loss of visual acuity or visual field, with or without eye pain.
- At least one of the following abnormalities: relative afferent pupillary defect, a nerve fiber bundle visual field defect, abnormal visual evoked potential.

Patients were excluded if they showed any evidence of compressive, vascular, toxic, metabolic, infiltrative, or hereditary optic neuropathy. We also excluded those who had retinal lesions or other causative ocular diseases [18].

Patients with unilateral or bilateral relapsing ON (RON), bilateral ON occurring at the same time (BON), or isolated unilateralON without relapse for 1 year (ION) were included in the study. The diagnosis of MS was confirmed using the 2010 revisions to the McDonald Criteria [19]. And the diagnosis of NMO must meet revised diagnostic criteria of Wingerchuk in 2006 [20]. Diagnosis of a rheumatologic disease or syndrome was according to international classification criteria, such as Sjögren’s syndrome [21], systemic lupus erythematosus (SLE) [22], ankylosing spondylitis (AS) [23], Wegner’s granulomatosis (WG) [24], anticardiolipin antibody syndrome (ACA) [25], rheumatoid (RM) [26], and Behcet’s disease (BD) [27].

Exclusion criteria comprised the presence of significant refractive errors (3D of spherical equivalent refraction or 2D of astigmatism), intraocular pressure of 21 mmHg or higher, systemic conditions that could affect the visual system, a history of ocular trauma or concomitant ocular diseases, including a history of media opacification, ocular pathologies affecting the cornea, lens, retinal disease, glaucoma, or laser therapy, retina diseases. All patients in the study groups could have one episode of ON more than 6 months before the study inclusion time point. The exclusion criteria also comprised hepatitis C infection, lymphoma, graft-versus-host disease, lymphoma, human T-lymphotropic virus Type I infection, human immune deficiency virus infection, and previous head or neck radiation. However, patients who were prescribed medications that might cause dry eye or dry mouth were not excluded.

Laboratory and radiological results were recorded. Serum was drawn for ANA, SSA/SSB, RF, ACLs, anti-β2-glycoprotein I (β2-GPI), A-ds DNA, and AQP4-Ab at the Examination Center for Biomedical Research of PLAGH. All serum samples were analyzed for the presence of AQP4-IgG antibodies by an extracellular live cell–staining immunofluorescence technique using transiently transfected AQP4-expressing cells as previously described [28].

The following magnetic resonance imaging (MRI) sequences of the brain/orbit were acquired using a 3 Tesla scanner (GE, USA), post-contrast T1-weighted conventional spin-echo (TR = 680 ms; TE = 14 ms, FOV = 24, slice thickness = 3.0, interleaved) 5 min after the intravenous administration of 0.1 mmol/kg GadopentetateDimeglumine. The treatments received by the patients were carefully documented. The Optic Neuritis Registry Forms and examination of eyes were completed by the resident neuro-ophthalmologist at PLAGH. Retinal nerve fiber layer thickness (RNFLT) was determined by optical coherence tomography (OCT). All OCT examinations were carried out using a high-definition spectral-domain optical coherence tomography (HD-OCT) (Cirrus HD-OCT; Carl Zeiss Meditec Inc., Dublin, CA). Both the Macular Cube 512×128 scan and RNFL measurement by the Optic Disc Cube 200×200 protocol were performed on all eyes [29]. All the data were analyzed using the Statistical Package for Social Sciences version 19.0 (SPSS Inc, Chicago, IL, USA).

### Ethics Statement

This study was approved by the People’s Liberation Army General Hospital Ethics Committee and was conducted following the Declaration of Helsinki in its currently applicable version. All individuals voluntarily participated in the study after a thorough oral and written information procedure. Oral and written consents were obtained from all participants.

## Results

### Cohort Demographics

Our cohort included 190 patients (302 eyes) with ON. All patients underwent clinical diagnosis, autoantibody detection, OCT examination, and orbit/brain MRI. autoantibodies were detected in all patients. There were 79 (40%) patients with an unilateral or bilateral relapsing ON including 134 eyes, whereas 60 patients (31.58%) had a history of unilateral ON including 60 eyes, and 54 patients (28.42%) having bilateral ON including 108 eyes. Fourteen patients were relapsing to NMO in the RON group, 6 patients in the BON group, and 3 patients in the ION group. Demographic and ON disease characteristics identified via medical record review were presented in Table 1. RON patients relapsed with a higher frequency to NMO. In the RON patients, there were 33 patients had their second episode (n = 33), 22 in third (n = 22), 10 in forth (n = 10), 6 in fifth (n = 6), 2 in sixth (n = 2), 2 in seventh (n = 7) and 1 in ninth (n = 1) of ON at the time of blood sampling.

**Table 1:** Epidemiologic and Disease Characteristics of Patients with three types of ON.

	RON	BON	ION	p1	p2	p3
Number, n	76	54	60			
Sex (Female/Male)	59/17	34/20	42/18			
Age, mean ±SD, y	39.82±16.05	39.33±13.80	37.51±14.85			
Years since disease onset, mean ±SD, y	2.26±4.17	1.46±1.46	1.22±0.56			
Intraocular pressure, mean ±SD, mmHg	14.25±6.12	15.2±3.74	13.96±5.13			
Eyes with optic neuritis history, n	134	108	60			
Follow up time,mean ±SD, months	34.42±35.97	26.42±26.47	20.04±8.089			
Relape to MS, n, %	4(5.26%)	1(1.85%)	1(1.67%)			
Relape to ADEM, n, %	0(0.00%)	0(0.00%)	0(0.00%)			
Relape to NMO, n, %	14(18.42%)	6(11.11%)	3(0.50%)	0.255	0.019*	0.390

* = P<0.05; ** = P<0.01. P1 = RON vs BON; P2 = RON vs ION; P3 = BON vs ION;

### Autoantibodies and CTDs in ON

Frequencies of autoantibodies and CTDs were assessed in RON, BON, and ION patients. Detailed results of autoantibody testing were presented in Table 2. There were in total 78 (35.79%) patients with abnormal autoantibodies. Autoantibodies were found in 39 (51.31%) RON patients, 21 (38.89%) BON patients, and 8 (13.33%) ION patients. The detection of autoantibodies by ON subtypes was also assessed. There were 20 (26.32%) patients, 11 (20.37%) patients, 3 (5.00%) patients, in the RON group, BON group, and ION group, respectively, with ANA titers equal to or greater than 1∶160. SSA or SSB was positive in 23 (30.26%) RON patients, 2 (3.70%) BON patients, and 3 (5.00%) ION patients, respectively. ACL orβ2-GPI were found in 9 (11.84%) patients with RON, 6 (11.11%) with BON, and 5 (8.33%) with ION. Anti-ds DNA was detected in 3 (3.95%) RON patients, 1 (1.85%) BON patient, and 0 (0.00%) ION patients. RF was found in 5 (6.58%) RON patients, 0 (0.00%) BON patients, and 0 (0.00%) ION patients. AQP4 antibodies were found in 10 (13.16%) RON patients, 3 (5.56%) BON patients, and 0 (0.00%) ION patients.

**Table 2:** Detection of autoantibodies in patients with ON.

	RON (n = 76)	BON (n = 54)	ION (n = 60)	*p*1	*p*2	*p*3
ANA(1∶160)	7 (9.21%)	3 (5.56%)	1 (1.67%)			
ANA(1∶320)	8 (13.33%)	6 (11.11%)	1 (1.67%)			
ANA(1∶640)	3 (3.95%)	0 (0.00%)	1 (1.67%)			
ANA(1∶1000)	2 (2.62%)	2 (3.70%)	0 (0.00%)			
	20 (26.32%)	11 (20.37%)	3 (5.00%)	0.432	<0.001**	0.013**
SSA	18 (22.78%)	2 (3.70%)	2 (2.25%)			
SSB	0 (0.00%)	0 (0.00%)	0 (0.00%)			
SSA+SSB	5 (6.58%)	0 (0.00%)	1 (1.67%)			
	23 (30.26%)	2 (3.70%)	3 (5.00%)	<0.001**	<0.001**	1.00
ACL	2 (2.63%)	0 (0.00%)	1 (1.67%)			
β2-GPI	5 (6.58%)	5 (9.26%)	1 (1.67%)			
ACL+β2-GPI	2 (2.63%)	1 (1.85%)	3 (3.37%)			
	9 (11.84%)	6 (11.11%)	5 (8.33%)	0.64	0.15	0.63
A-AQP-4 Ab(>1∶10)	10 (13.16%)	3 (5.56%)	0 (0.00%)	0.044*	0.01**	0.103
A-ds DNA	3 (3.95%)	1 (1.85%)	0 (0.00%)	0.868	0.333	0.958
RF	5 (6.58%)	0 (0.00%)	0 (0.00%)	0.144	0.117	N/A
Total	39 (51.31%)	21 (38.89%)	8 (13.33%)	0.16	<0.001**	0.002**

ANA: antinuclear antibody, SSA and SSB: extractable nuclear antigen antibodies, RF:rheumatoid factor,ACL: anticardiolipin antibody, AQP-4: aquaporin-4; A-ds DNA: anti-double-stranded DNA antibody;

* = *p*<0.05; ** = *p*<0.01. *p*1 = RON vs. BON; *p*2 = RON vs. ION; *p*3 = BON vs. ION.

Statistical comparisons were made among ON subtypes (Table 2). Patients in the RON group had a higher frequency of autoantibodies detection (51.31% vs. 13.33%, p2<0.001), including a higher frequency of ANA (26.32% vs. 5.00%, p2<0.001), SSA/SSB (30.26% vs. 5.00%, p2<0.001) and AQP4 antibodies (7.89% vs. 0.00%, p2 = 0.01), than patients in the ION group. Patients in the BON group had a higher frequency of autoantibodies detection (38.89% vs. 13.33%, p3 = 0.002) than the ION group, including a higher frequency of ANA (20.37% vs. 5.00%, p3 = 0.013). Patients in the RON group had a higher frequency of SSA/SSB (30.26% vs. 3.70%, p1<0.001) than patients in the BON group. (Table 2).

There were 26 patients who fulfilled clinical classification criteria for SS (15 patients), SLE (4), ACA(1), AS(2), RM(1), WG(2), and BD(1). (Table-3) The RON group showed a higher tendency to occur together with CTD than the ION group (p = 0.007). (Table-3) Comparisons were also made among ON subtypes, RON patients had SS more frequently than patients in the BON and ION groups, and the differences between the three groups were statistically significant. (Table 3).

**Table 3:** Diagnosis of different rheumatologic diseases.

	RON (n = 76)	BON (n = 54)	ION (n = 60)	*p*1	*p*2	*p*3
AS	1 (1.32%)	1 (1.85%)	0 (0.00%)	1.00	1.00	0.958
RM	0 (0.00%)	0 (0.00%)	1 (1.67%)	N/A	0.905	1.00
BD	0 (0.00%)	1 (1.85%)	0 (0.00%)	0.863	N/A	0.958
WG	0 (0.00%)	2 (3.70%)	0 (0.00%)	0.333	N/A	0.430
SLE	3 (3.95%)	1 (1.85%)	0 (0.00%)	0.868	0.333	0.958
ACA	1 (1.32%)	0 (0.00%)	0 (0.00%)	1.00	1.00	N/A
SS	11 (14.47%)	2 (3.70%)	2 (3.33%)	0.04*	0.028*	1.00
Total	16 (21.05%)	7 (12.96%)	3 (5.00%)	0.233	0.007**	0.242

AS: Ankylosing spondylitis; RM: Rheumatoid; BD: Behcet’s disease; WG: Wegner’s granulomatosis; SLE: Systemic lupus erythematosus; ACA: Anticardiolipin antibody syndrome; SS: Sjogren’s syndrome.

* = *p*<0.05; ** = *p*<0.01. *p*1 = RON vs. BON; *p*2 = RON vs. ION; *p*3 = BON vs. ION.

### Sjögren’s Syndrome Symptoms with ON

A diagnosis of Sjögren’s syndrome was performed according to European Study Group on Classification Criteria for Sjögren’s syndrome [14]. In this study, ON occurring with SS had been summarized in Table 4. Fourteen females and 1 male were judged eligible for the study. Eleven patients were included in the RON group, 2 in the BON group, and 1 inthe ION group. One patient was alsoAQP4 antibodies positive (titer = 1∶100). Three (20%) patients relapsed to NMO. Long T2-weighted image post-gadolinium fat-saturated T1-weighted imaging of optic nerve demonstrated the marked enhancement in 10 patients. Seven patients only received methylprednisolone pulse therapy, 8 patients received an initial high-dose immunosuppressive and methylprednisolone pulse therapy. After therapy, the patients who were given immunosuppressive therapy had better vision acuity recovery, and a reduced number of recurrences. Statistical comparisons were not made due to the small sample size.

**Table 4:** Disease Characteristics of ON Patients combined with SS.

Number	Sex	Age (year)	ANA	SSA/SSB	Years since disease onset	CSF-abnormal OCB and IgG	Times of relapes	AQP-4 antibody	Relapse to NMO	Orbtic MRI	Treatment	Visual acuity after treatment	
												R	L
1	Female	15	1∶640	SSA+SSB	19 months	Neg	2	Neg	N	high signal on T2	G+IS	1.0	1.0
2	Female	21	1∶320	SSA	36 months	Neg	5	Neg	N	None	G+IS	0.1	1.0
3	Female	58	1∶640	SSA	21 months	Neg	2	Neg	N	high signal on T2	G+IS	0.3	1.0
4	Female	48	1∶320	SSA	36 months	Neg	2	Neg	N	None	G+IS	0.1	1.0
5	Female	34	1∶1000	SSA+SSB	15 months	Neg	2	Neg	N	enhancement.	G+IS	1.0	1.0
6	Female	32	1∶640	SSA+SSB	14 months	Neg	2	Neg	N	enhancement.	G+IS	1.0	1.0
7	Female	41	1∶320	SSA	15 months	Neg	0	Neg	NMO	enhancement.	G	FC/10cm	
8	Female	56	1∶160	SSA	11 years	Neg	5	Neg	N	None	G+IS	0.8	0.6
9	Female	21	1∶320	SSA+SSB	12 months	Neg	2	1∶00	NMO	None	G	0.15	0.2
10	Female	49	1∶320	SSA+SSB	5 years	Neg	4	Neg	N	None	G	0.5	FC/30cm
11	Female	48	1∶160	SSA+SSB	7 months	Neg	0	Neg	N	enhancement.	G	FC/10cm	
12	Female	42	1∶640	SSA	12 months	Neg	0	1∶00	NMO	enhancement.	G	0.5	0.8
13	Female	55	1∶320	SSA	13 months	Neg	2	Neg	N	high signal on T2	G	1.0	0.12
14	Female	46	1∶1000	SSA	14 months	Neg	3	1∶00	N	high signal on T2	G	0.1	0.15
15	Male	49	1∶1000	SSA	8 month	Neg	0	Neg	N	enhancement.	G+IS	0.3	0.5

Neg: negative; N: not relapse to NMO; None: none abnormal signals; G:glucocorticoid; IS: immunosuppressive.

In this study, patients with and without SS, (Table 5) but SSA/SSB positive were compared. OCT was used to test for atrophy of the optic nerve; AQP4 antibodies were also detected. There were no significant differences between the two groups in RNFLT, number of relapses, AQP4 antibodies, and relapsed to NMO. Differences between the two groups were found with respect to teeth symptoms, and in minor salivary gland biopsy.

**Table 5:** Differences between patients with SS and positive SSA/SSB but not SS diagnosis.

	SS(+) n = 15	SS(−) n = 13	*p*
Years since disease onset	2.27±2.81	4.17±7.58	0.29
Times of relapse	2.07±1.86	1.86±1.79	0.76
Sex (female:male)	(14∶1)	(12∶1)	0.92
Age (year)	41.00±13.54	43.08±16.94	0.73
RNFL (µm) Average thickness	71.90±17.67	69.58±13.62	0.64
Superior quadrant	85.00±26.40	79.68±20.27	0.48
Inferior quadrant	86.28±27.42	83.47±27.95	0.75
Nasal quadrant	61.05±11.62	62.89±12.27	0.63
Temporal quadrant	51.29±14.23	48.89±11.37	0.56
AQP-4 antibody	3 (20.00%)	3 (23.07%)	1.00
Relapse to NMO	3 (20.00%)	3 (23.07%)	1.00
Ocular symptom	10 (66.67%)	5 (38.46%)	0.255
Oral symptom	12 (80.00%)	10 (76.92)	1.00
Gland symptom	7 (46.67%)	0 (0.00%)	0.007*
Teeth symptom	7 (46.67%)	0 (0.00%)	0.007*

* = p<0.05; ** = p<0.01.

### Comparison of Patients with and without Positive SSA/SSB

The differences of SSA/SSB positive and SSA/SSB negative patients were summarized in Table 6. A total of 68 patients had abnormal autoantibodies, and 28 (41.18%) patients were SSA/SSB (+), and the remaining 40 (58.82%) were SSA/SSB (-). OCT was used to test for atrophy of the optic nerve, and AQP4 antibodies were also detected. No statistically significant differences were found between the two groups with respect tothe number of eyes with ON, course of the disease, RNFLT, number of relapses, AQP4 antibodies, and relapsed to NMO.

**Table 6:** Differences between patients with positive SSA/SSB and negative SSA/SSB.

	SSA/SSB(+)	SSA/SSB(−)	*p*
Number of patients, n	28	40	
Years since disease onset, mean ±SD, y	3.16±1.79	2.67±2.79	0.62
Times of relapse, mean ±SD, n	1.96±5.58	1.41±1.74	0.23
Sex (female/male)	(26/2)	(30/10)	
Eyes with ON history, n	52	56	
Age (year)	44.5±14.96	36.56±16.87	0.18
RNFL (µm) Average thickness	70.80±15.72	69.78±17.30	0.8
Superior quadrant	82.48±23.54	78.44±22.06	0.46
Inferior quadrant	84.95±27.35	84.87±31.74	0.99
Nasal quadrant	61.93±11.81	61.50±12.41	0.88
Temporal quadrant	50.15±12.85	54.06±14.19	0.23
Relapse to NMO	6 (21.43%)	8 (20%)	0.88

* = *p*<0.05; ** = *p*<0.01.

## Discussion

Frequencies of autoantibodies and CTDs were assessed in RON, BON, and ION patients. In this study, RON and BON patients had higher frequencies of Auto-Absband CTDs than ION patients; results with respect to AQP4 antibodies were identical. The RON group was significantly different from the other groups in RF, ANA, and SSA/SSB. SS was the most common CTD in RON patients, and was different from the other groups. BON patients had a higher frequency of ANA than patients in the ION group, but were not significantly different from ION patients with respect to other Auto-Abs and CTDs. We also analyzed the differences in clinical characteristics between patients with SS and those not meeting the diagnostic criteria for SS but who were SSA/SSB positive. No statistically significant difference between them was found in terms of sex, age, number of relapses, years since disease onset, rate of relapse to NMO, and beingAQP4 antibodies positive.

RON and BON patients had a higher frequency of having Auto-Absband CTDs than ION patients. From these results, we noted that SSA/SSB and SS was more common in the RON group (SSA/SSB 23 (30.26%) and SS 11 (14.47%)), and there was a higher frequency of AQP4 antibodies, 10 (13.16%) in the RON group than in the other groups. This finding was much different from previous studies in MS that identified SSA/SSB [30] and the finding of SSA/SSB in this study was very similar to NMO [31]. Moreover, the incidences of BON and ION with SSA/SSB were similar to MS in previous studies [32]. SS was a systemic autoimmune disease that presents with sicca symptomatology of the main mucosal surfaces and gland inflammation, and often presented with other immunological diseases [33]. In recent studies, it had been demonstrated that SS was related to central nervous system diseases, including MS and NMO [34]. NMO could coexist with CTDs, particularly SS, but this association was rare with MS [35], the prevalence of MS with SS was only ranging from 0% to 3.3% [36]. In the RON group,there were 11 (14.47%) patients diagnosed with SS, the frequency was higher than reported in previous studies in MS, and the rates of BON 2 (3.70%) and ION 2 (3.33%) with SS were similar as reported before [37], [38]. Therefore, we concluded that RON was more like NMO, and BON and ION were more like MS.

There were only few studies researching the clinical characteristics of ON with positive auto-Abs (with and without SSA/SSB). We compared the differences between ON with and without SSA/SSB; there were no statistically significant differences between them with respect to sex, age, number of relapses, years since disease onset, and eyes with ON history. OCT was used to evaluate changes in the optic nerve, because it was more sensitive and objective than other methods for assessing the severity of atrophy of the optic nerve [39]. Based on the results, it seemed that SSA/SSB occurred more frequently with RON, but it had no effect on the severity and prognosis of ON. There were no statistically significant differences between the rate of relapse to NMO and AQP4 antibodies positive. AQP4 antibodies were a specific biomarker indicating NMO, with patients with AQP4 antibodies often having a prognosis of severe vision loss with ON [40]. In previous studies, relapse of ON (14.8%) was more likely in Chinese patients, being AQP4antibodies seropositive [41], and we noted similar results (13.16%) in this study. The results indicated that RON was more common than BON and ION with AQP-4 antibody, and there were more relapses to NMO. AQP4 antibodies were important in RON and it was useful on disease progression to NMO. Matiello et al. showed that [42] AQP4 IgG antibodies seropositivity predicted poor visual outcome and development of NMO. And A multicentre study of 175 patients in Germany found that a visual acuity of ≤0.1 was more frequent during acute optic neuritis (ON) attacks among AQP4 antibodies seropositives [43]. So, it was necessary for RON patients to test AQP4 antibodies.

MS and NMO were the most common diseases studied with respect to SS involvement in the central nervous system [44], [45]. There were only few studies about ON combined with SS. Here, we summarized the clinical characteristics of ON patients who also met the criterion for SS. In this study, there were 1 male and 14 female patients, the reason for this imbalance beingthat males received a diagnosis of a CTD less frequently than females [46], [47]. None of the patients showed abnormal OCB and IgG in CSF, in contrast to MS patients [48]. All the patients were examined by MRI; however, there were no significant differences when compared with other ON patients. In our opinion, MRI lacked the specificity to diagnose SS-ON. We compared data from seropositive SS, but the criteria was not met. Patients with coexisting SS did not significantly associate with axonal loss of the optic nerve from just SSA/SSB seropositive patients. Differences between them included the sicca complex (xerostomia, xerophthalmia, decayed teeth, and minor salivary gland biopsy), which were the features of SS. Immunosuppressive or immunomodulatory drugs seemed useful for improving vision recovery. Statistical analysis was not done, because of the sample size,whereas previous studies that involved other central nervous system diseases with SS had reported that immunosuppressive and immunomodulatory treatment could reduce the number of relapses and improve recovery [49], [50].

Antibody status might be different at the first episode or at a subsequent episode, so it is more significant to include longitudinal analysis of these samples. However, theinitial diagnoses of many RON patients weremade in other hospitals. Due to the high cost, most of them had not completed autoantibodies array before came to our hospital. Therefore this study is a cross sectional analysis. Nevertheless, we had noted this interesting point, longitudinal analysis will be made when the clinical follow up time is enough.

Myelin oligodendrocyte glycoprotein (MOG) antibodies were also an important antibody in demyelination of the central nervous system. Patients with MS show a greater proliferative response to myelin oligodendrocyte glycoprotein (MOG) antibodies compared to other antigens in peripheral blood lymphocytes [51]. MOG antibodies can be detected in patients with ON and most likely do play a role in the disease process in contrast to the other autoantibodies apart from AQP-4 detected. MOG antibodies seropositive in patients of NMOSD have fewer attacks, and better recovery than patients with AQP4 antibodies or patients seronegative for both antibodies [52]. High MOG-IgG levels were also significantly detected in patients with RON who had MRI findings ranging from normal to optic nerve swelling. Therefore, anti-MOG antibody may be a new tool that can be used to separate ON patients from those who will eventually develop MS or NMO [53]. In our study we did not include the assessment of MOG antibodies, because of small samples and need for substantial follow-up times. It will be assessment in future studies.

## Conclusion

RON and BON were more likely to occur with abnormal autoantibodies. Furthermore, SSA/SSB and SS were more common in RON patients, and RON patients were more likely to also have AQP-4 antibody and relapse to NMO. But compared with other autoantibodies, SSA/SSB detected in patients were not significantly associated with disease characteristics or severity.
